# The magic bullet: Niclosamide

**DOI:** 10.3389/fonc.2022.1004978

**Published:** 2022-11-21

**Authors:** Haowen Jiang, Albert M. Li, Jiangbin Ye

**Affiliations:** ^1^ Department of Radiation Oncology, Stanford University School of Medicine, Stanford, CA, United States; ^2^ Cancer Biology Program, Stanford University School of Medicine, Stanford, CA, United States; ^3^ Stanford Cancer Institute, Stanford University School of Medicine, Stanford, CA, United States

**Keywords:** niclosamide, mitochondrial uncoupler, metabolism, epigenetics, anti-tumor effect, oncogenic pathways, tumor suppressors, magic bullet

## Abstract

The term ‘magic bullet’ is a scientific concept proposed by the German Nobel laureate Paul Ehrlich in 1907, describing a medicine that could specifically and efficiently target a disease without harming the body. Oncologists have been looking for a magic bullet for cancer therapy ever since. However, the current therapies for cancers—including chemotherapy, radiation therapy, hormone therapy, and targeted therapy—pose either pan-cytotoxicity or only single-target efficacy, precluding their ability to function as a magic bullet. Intriguingly, niclosamide, an FDA-approved drug for treating tapeworm infections with an excellent safety profile, displays broad anti-cancer activity in a variety of contexts. In particular, niclosamide inhibits multiple oncogenic pathways such as Wnt/β-catenin, Ras, Stat3, Notch, E2F-Myc, NF-κB, and mTOR and activates tumor suppressor signaling pathways such as p53, PP2A, and AMPK. Moreover, niclosamide potentially improves immunotherapy by modulating pathways such as PD-1/PDL-1. We recently discovered that niclosamide ethanolamine (NEN) reprograms cellular metabolism through its uncoupler function, consequently remodeling the cellular epigenetic landscape to promote differentiation. Inspired by the promising results from the pre-clinical studies, several clinical trials are ongoing to assess the therapeutic effect of niclosamide in cancer patients. This current review summarizes the functions, mechanism of action, and potential applications of niclosamide in cancer therapy as a magic bullet.

## Introduction

In 1907, the German Nobel Laureate Paul Ehrlich conceived the pioneering concept of the “magic bullet,” a medicine that specifically targets disease without causing harm to healthy tissues (1). Based on this theory, he identified salvarsan as the first “magic bullet” for syphilis in 1909. Likewise, oncologists have sought a magic bullet for cancer therapy, culminating in the discovery of chemotherapy (2). However, generations of oncologists interpreted the magic bullet as a compound that could target a single protein encoded by a crucial oncogene, without proper consideration of the fact that cancer is a systemic disease that is not driven by a single driver/mutation (1). In fact, given the genetic heterogeneity of tumors, targeting the gene product(s) of any single mutation would lead to the selective outgrowth of a cancer cell population carrying other mutations, resulting in drug resistance and relapse (3). Thus, targeted therapy and other current cancer therapies that pose pan-cytotoxicity in patients, such as chemotherapy and radiation therapy, do not qualify as magic bullets. A true “magic bullet” for cancer treatment remains to be identified.

According to Otto Warburg, the inhibition of mitochondrial respiration leading to enhanced lactate production from glycolysis, namely the Warburg effect, is the primary cause of tumorigenesis (4, 5). The electron transport chain (ETC) coupled to ATP synthesis represents the core function of mitochondrial respiration. Based on Warburg’s theory, we hypothesize that activating the ETC could reverse the Warburg effect and inhibit tumorigenesis. A potential candidate is the mitochondrial uncoupler niclosamide, an FDA-approved anthelmintic medicine that has been used to treat tapeworm infestations for nearly 50 years ^6^. Recently, a number of studies and clinical trials have aimed to repurpose niclosamide for Covid-19 and cancer treatment (6, 7). Accumulating evidence indicates that niclosamide is a pleiotropic compound that targets multiple biological processes and signal pathways. Because niclosamide shuttles electrons across the mitochondrial inner membrane to activate the ETC, niclosamide reprograms intracellular metabolism (8), which can impact cellular epigenetic regulation at the transcriptional, translational, and post-translational levels (9, 10). Furthermore, the ability of niclosamide to modify the global epigenetic landscape through metabolic reprogramming (8) may explain its ability to simultaneously inhibit oncogenic signaling pathways and activate tumor suppressor signaling pathways. The fact that a modulator of metabolism, such as niclosamide, inhibits tumorigenesis through potentially pleiotropic mechanisms further validates Warburg’s hypothesis: the primary cause of tumorigenesis is metabolic reprogramming.

## The discovery, nomenclature, formula and structure of niclosamide

**Figure 1:**
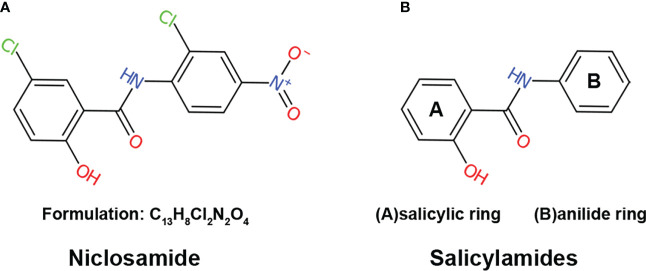
The structure of niclosamide **(A)** The structure and formulation of niclosamide. **(B)** The structure of salicylamides, which are weakly acidic phenolic compounds consisting of two basic chemical structures: a salicylic acid ring and an anilide ring.

Niclosamide, also known as Bayluscide, was first discovered in the Bayer chemotherapy research laboratories in 1958 (11) through screening chemical compounds against the aquatic pulmonated gastropod mollusk Biomphalaria glabrate, an intermediate host for the human parasitic trematode Schistosoma mansoni. As a secondary carboxamide that goes by the name of 5-Chloro-N-(2-chloro-4-nitrophenyl)-2-hydroxybenzamide in the IUPAC nomenclature system, niclosamide is a product formed through the condensation of the carboxy group of 5-chlorosalicylic acid with the amino group of 2-chloro-4-nitroaniline (**Figure 1A**). The molecular formulation of niclosamide is C_13_H_8_C_l2_N2O4 with a molecular weight of 327.12 Dalton (Da). Niclosamide is considered thermally stable, with hydrolysis only happening by boiling in concentrated alkalis or acids (12). 

## Applications of niclosamide as an anthelmintic drug

Niclosamide is a widely used anthelmintic drug in the treatment of parasitic infections. It was approved by the FDA in 1982 and listed in the World Health Organization’s list of essential medicines (13, 14). It is generally taken at a 2g single dose for adults and 1-1.5g single dose for the children^4^. For *D. latum*, *T. saginata*, *D. caninum*, and *T. solium*, a single dose of niclosamide is effective. Because niclosamide is not effective against mature *H. nana* cysts, effective treatment regimens require repeated daily doses for 1 week to completely eradicate the infection (13). In humans and animals, niclosamide is partially absorbed in the intestinal canal and rapidly eliminated by the kidney (11). The original pharmacokinetics study showed that the maximal serum concentration can reach 0.25-6.0ug/ml (0.76-18.34 µM) following administration of a single 2g dose (11). The native form of niclosamide, along with its derivatives 2’,5-dichloro-4’-aminosalicylanilide and 2’,5-dichloro-4’-acetaminosalicylanilide, has been shown to be completely eliminated from the human body within 1-2 days (11). Overall, niclosamide shows a significant anthelmintic effect along with a strong safety profile and tolerability in humans.

## Mechanism of action: Mitochondrial uncoupling

Mitochondrial uncoupling is a process that dissipates the proton gradient across the inner mitochondrial membrane, inhibiting ATP synthesis and activating the ETC to promote NADH oxidation (15, 16). Niclosamide is a derivative of salicylamides, a class of potent mitochondrial uncouplers (17–20). Salicylamides are weakly acidic phenolic compounds consisting of two basic chemical structures: a salicylic acid ring and an anilide ring (**Figure 1B**). In general, drugs with uncoupling properties possess three characteristics: an acid dissociable group, a bulky hydrophobic moiety, and strong electron-withdrawing group (21). In the case of salicylamides, the salicylic acid ring and anilide ring serve as the acid-dissociable group and bulky hydrophobic moiety, respectively, while the amide group is the electron-withdrawing group (22).

**Figure 2:**
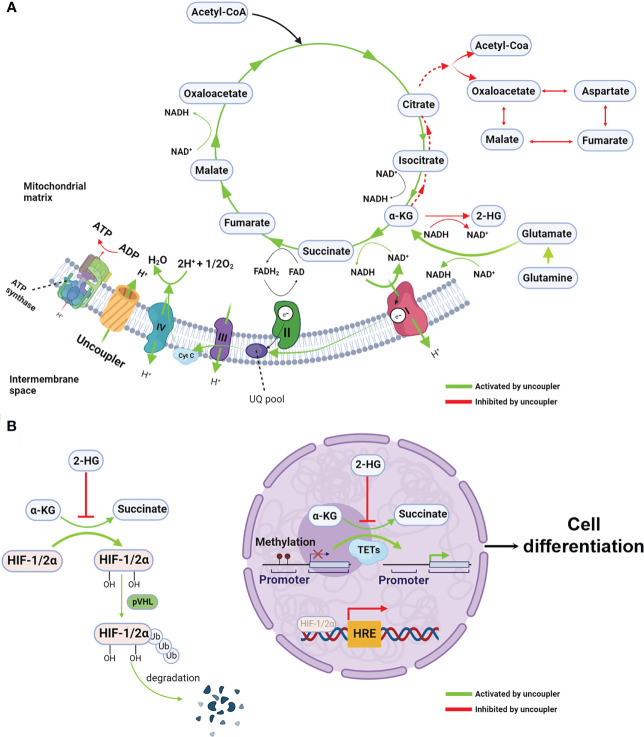
Mitochondrial uncoupling reprograms metabolism and epigenetic landscape **(A)** Mitochondrial uncouplers dissipate the proton gradients which are essential to ATP synthesis, resulting in reduction of ATP/ADP ratio. When proton gradient reduce, the electron transfer chain, particularly complex I, are activated, leading to increased intracellular redox NAD^+^/NADH ratio. Given the NAD^+^/NADH ratio is the major driving force for TCA cycle, the oxidative TCA cycle and glutaminolysis are accelerated. Because the chemical equilibrium of many metabolites pair such as α-KG/2-HG and pyruvate and lactate (not show in the figure) are dictating by NAD^+^/NADH ratio. Thus, increased NAD^+^/NADH mediated by mitochondrial uncoupler shift the equilibrium from 2-HG to α-KG, resulting in increased α-KG/2-HG ratio. In the other hand, opposite to the oxidative TCA cycle, the reductive TCA cycle particular reductive carboxylation is inhibited by mitochondrial uncoupler. **(B)** The increased α-KG/2-HG ratio activates the α-KG-dependent dioxygenases such as TET and PHD, leading to DNA demethylation and HIFs protein degradation. These epigenetic rewiring activate the expression of differentiation makers and repress the stemness genes, consequently, cell differentiation. Created with >BioRender.com.

Structural studies have determined that the formation of a six-membered hydrophobic ring between a -NH in the aniline moiety and a phenolic -OH in the salicylic acid moiety by intramolecular hydrogen bonding contributes to the high hydrophobicity and structural stability important for uncoupler activity (21, 22). These chemical structures are absolutely essential for the mitochondrial uncoupling activity of salicylamides (22–25). For example, replacing the phenolic hydroxyl (-OH) group to a methyl (-CH_3_) of niclosamide is thought to abolish its mitochondrial uncoupling activity, resulting in a loss of anti-growth effect in both wild-type or p53-null cancer cells, suggesting that the antitumor effect of niclosamide relies on its uncoupling function (20). A signaling mechanism by which this effect is thought to be mediated involves niclosamide decreasing the mitochondrial potential to inhibit ATP synthesis (**Figure 2A**), leading to the activation of AMPK and the induction of either cell cycle arrest or apoptosis (15, 18–20). Nonetheless, a potential downside exists; namely, the hydrophobic properties of mitochondrial uncouplers may limit their bio-availability as drugs.

A potential solution to the aforementioned challenge is niclosamide ethanolamine (NEN), a salt form of niclosamide that also functions as a mitochondrial uncoupler with a superior safety profile and enhanced bioavailability (11, 26). Alasadi et al. reported that NEN treatment enhances pyruvate entry into mitochondria, and reduces glucose flux to the pentose phosphate pathway, serine synthesis, and lactate production (15). Recently, we discovered that NEN activates the ETC to boost NADH oxidation, thereby leading to an increased intracellular NAD^+^/NADH ratio and driving the TCA cycle forward. The NAD^+^/NADH ratio dictates the equilibrium of pyruvate/lactate and α-ketoglutarate (α-KG)/L-2-hydroxyglutarate (L2-HG) (27–29). Excessive lactate production is a hallmark of the Warburg effect, and 2-HG is a competitive inhibitor of α-KG-dependent dioxygenases such as DNA demethylase ten eleven translocation enzymes (TET) (30, 31). NEN treatment increases the intracellular pyruvate/lactate ratio, the α-KG/2-HG ratio, and total intracellular α-KG levels, leading to a reversal of the Warburg effect and the induction of cellular differentiation (**Figure 2A**). Consistent with these observations, NEN treatment induces promoter CpG island demethylation and epigenetic landscape remodeling (**Figure 2B**) (8). In neuroblastoma cells, many genes activated by NEN treatment are involved in neurogenesis, nervous system development and neuron differentiation. The NEN-upregulated genes are enriched in the favorable prognosis gene signatures, while the NEN-downregulated genes are more enriched in unfavorable prognosis gene signatures. Consistent to the prognosis gene signatures changes, NEN treatment not only reduced the tumor growth but also prolonged the survival for tumor bearing mice (8). In vivo, NEN treatment also effectively increased the NAD+/NADH ratio and reduced lactate and 2-HG levels in xenograft tumors (8).

Together, these data suggest that when the ETC is inhibited, a shift towards more Warburg-like metabolism leads to cell dedifferentiation, a consequence of global epigenetic remodeling rather than alterations within a single gene or a pathway. Thus, activating the ETC with mitochondrial uncouplers not only antagonizes the Warburg effect by promoting TCA cycling, but also redirects the cellular epigenome and transcriptome towards that of a differentiated state. This highlights the advantage mitochondrial uncouplers hold over other drugs: the ability to target many oncogenic pathways simultaneously.

## Common signaling targets of niclosamide

**Figure 3:**
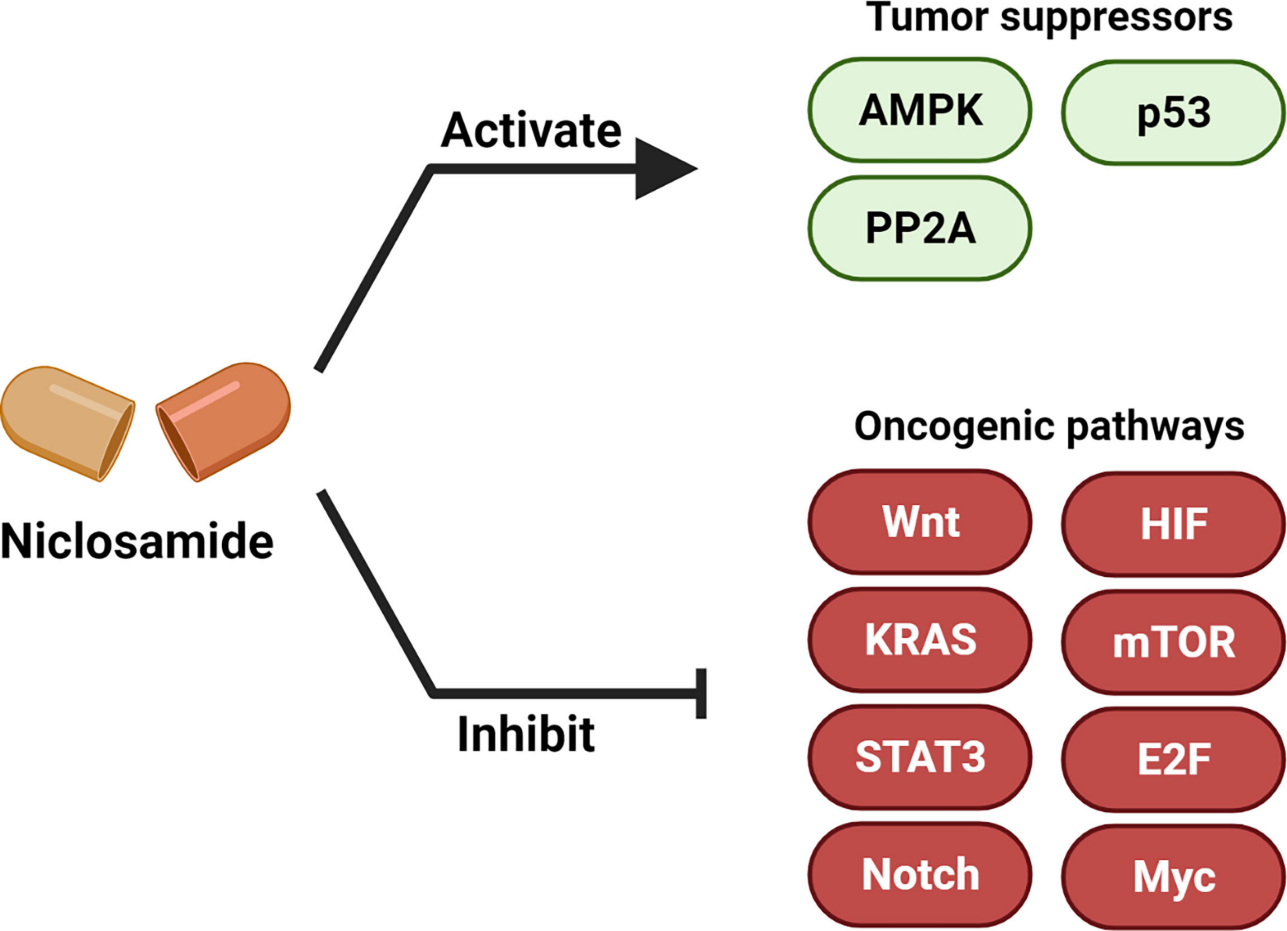
Niclosamide activates tumor suppressors and inhibits oncogenic pathways. Niclosamide has anti-tumor effect through inhibiting multiple oncogenic pathways such as Wnt/β-catenin, Ras, Stat3, Notch, E2F-Myc, NF-κB and mTOR, and activating tumor suppressor signaling such as p53, PP2A and AMPK. Created with BioRender.com.

Multiple studies have now demonstrated the anti-cancer efficacy of niclosamide (6, 32). In this section, we summarize the major oncogenic and tumor suppressor signaling pathways that are modulated upon niclosamide treatment (**Figure 3**, **Table 1**).

**Table 1:** Niclosamide activates tumor suppressor and inhibit oncogenic pathways.

	Target pathway	Effect of niclosamide	Cancer type	Reference
Oncogenic pathways	Wnt/β-catenin	Inhibition	sarcoma	(33)
			prostate	(34)
			breast	(34, 35)
			colon	(36–38)
			ovarian	(39)
			pancreas	(40, 41)
	KRAS	Inhibition	colon	(42)
			liver	(43)
			ovarian	(44)
	STAT3	Inhibition	prostate	(45, 46)
			lung	(47, 48)
			colon	(49, 50)
			breast	(51, 52)
			liver	(53)
	Notch	Inhibition	colon	(54)
			liver	(55)
	E2F	Inhibition	neuroblastoma	(8)
	N-myc	Inhibition	neuroblastoma	(8)
	c-Myc		ovarian	(56)
	NF-kB	Inhibition	ovarian	(44, 56)
			leukemia	(57, 58)
	mTOR	Inhibition	breast	(59)
			cervix	(60, 61)
			lung	(62, 63)
			ovarian	(64)
	HIF1α	Inhibition	colon	(65, 66)
			lung	(67)
			neuroblastoma	(8)
Tumor suppressors	p53	Activation	ovarian	(20)
			neuroblastoma	(8)
	AMPK	Activation	liver	(18, 68)
	PP2A	Activation	lung	(69)

### Oncogenic pathways

#### Wnt/β-catenin

The Wnt/β-catenin pathway is a developmental signaling pathway that regulates multiple key cellular biological processes including proliferation, migration, genetic stability, polarity, apoptosis, differentiation, and stem cell renewal (70, 71). The Wnt/β-catenin pathway is commonly dysregulated in many cancer types, leading to research into the role of WNT signaling in tumorigenesis and the subsequent development of various Wnt signaling inhibitors for cancer therapies. In the absence of Wnt ligands, cytosolic β-catenin is sequestered by its destruction complex APC, axis inhibitor (AXIN), casein kinase 1α (CK1α), and glycogen synthase kinase 3β (GSK3β) (72). Subsequently, phosphorylation of β-catenin by both CK1α and GSK3β marks itself with ubiquitination by E3 ligases β-transducin repeat–containing protein (βTrCP), resulting in proteasomal degradation (71, 72). Conversely, when extracellular Wnt protein binds to a heterodimeric complex of Frizzled receptors (FZD) and coreceptors low-density lipoprotein receptor-related proteins 5 and 6 (LRP5/6), the cytoplasmic tail of LRPs is phosphorylated, recruiting axis inhibition (AXIN) and the destruction complex to the cell membrane and activating dishevelled (DVL). The activated DVL represses the destruction complex of β-catenin, allowing the cytoplasmic accumulation and nuclear translocation of β-catenin. Subsequently, nuclear β-catenin interacts with T-cell factor/lymphoid enhancing factor (TCF/LEF) to induce the expression of specific target genes (71, 72).

**Figure 4:**
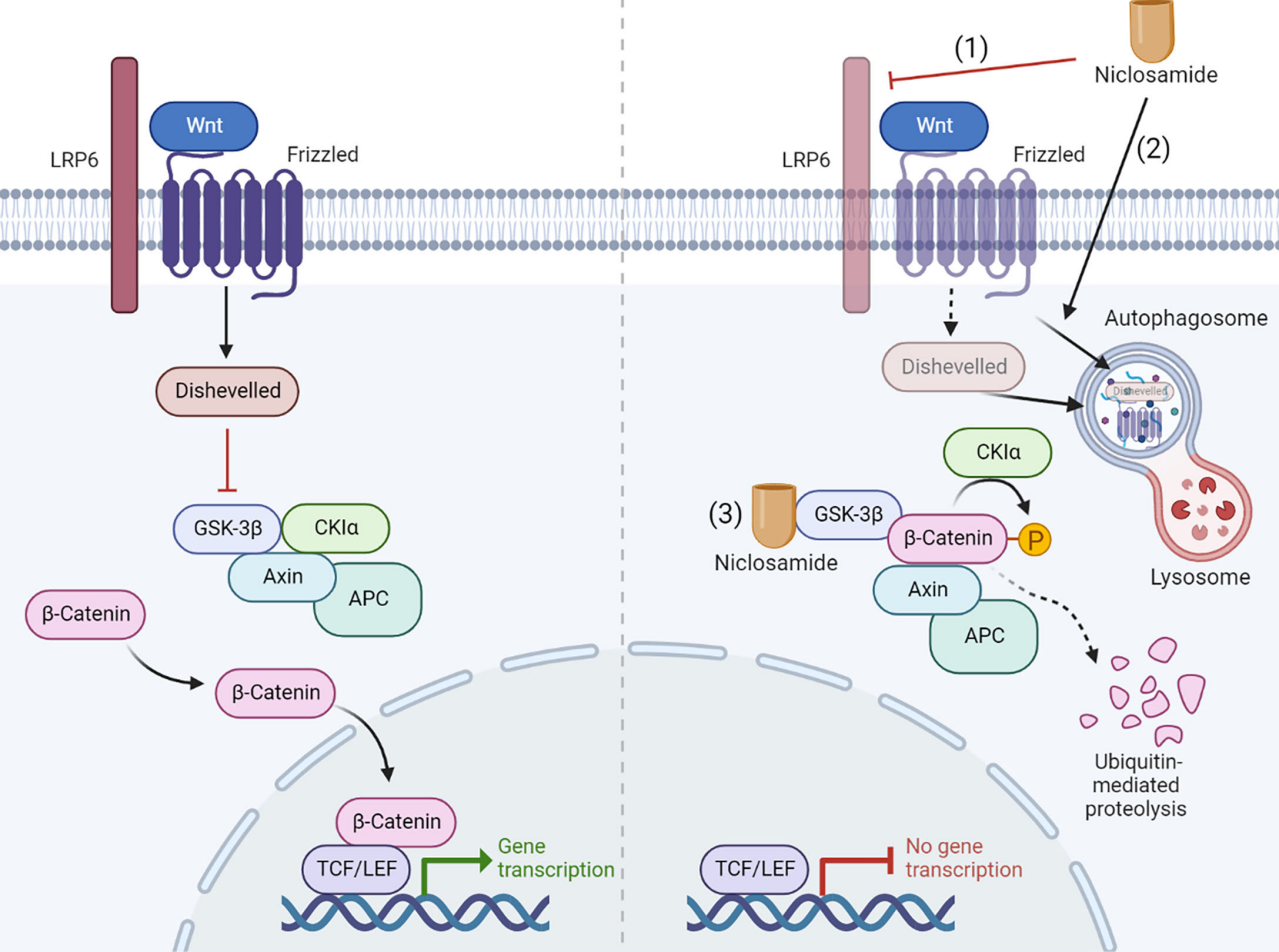
Niclosamide inhibits Wnt pathway through multiple mechanism. The Wnt pathway inhibition by niclosamide depends on multiple ways of action: (1) Niclosamide suppresses LRP6 expression. (2) Niclosamide promotes the degradation of Frizzled 1 and Dishevelled-2 through autophagy. (3) Niclosamide binds GSK3 directly, resulting in disruption of the Axin-GSK3 complex and attenuation of canonical Wnt activity. Created with BioRender.com.

Niclosamide inhibits Wnt/β-catenin signaling at multiple levels. Employing a primary imaged-based GFP fluorescence assay that uses Frizzled1 endocytosis as the readout to perform a high-throughput screen, Chen et al. reported that niclosamide downregulates Dishevelled-2 protein levels, antagonizing the Wnt3A-mediated induction of β-catenin and its downstream transcriptional activity (33). Ensuing studies have reported on the efficacy of niclosamide in targeting Wnt/β-catenin pathway in a wide spectrum of cancer types including prostate (34), ovarian (39), breast (34, 35), colorectal (36–38), pancreatic (41), and neuroblastoma (8). Niclosamide-driven Dishevelled-2 and Frizzled 1 degradation may also rely on the induction of autophagosomes (36, 38). Autophagosomes are double-membrane sequestering vesicles, originating from phagophores that engulf parts of the cytoplasm, eventually fusing with lysosomes to initiate substrate degradation (73). In support of this model, Frizzled 1 or β-catenin co-localizes with LC3, an autophagosome marker, in niclosamide-treated cells. Furthermore, niclosamide-mediated inhibition of Wnt/β-catenin signaling is rescued by the autophagosome inhibitor 3-MA and is attenuated in autophagy-deficient ATG5^−/−^ MEF cells (38). At the signaling receptor level, niclosamide suppresses LRP6 expression and phosphorylation, leading to a block in β-catenin stabilization induced by Wnt3A without affecting the expression level of Dishevelled-2 (34, 39). Niclosamide was also reported to bind GSK3 directly, resulting in disruption of the Axin-GSK3 complex and attenuation of canonical Wnt activity (37). A recent study reported that niclosamide increases GSK-3β phosphorylation to promote the ubiquitin-mediated degradation of β-catenin (41). The mechanism of Wnt pathway inhibition by niclosamide is summarized in **Figure 4**.

#### K-Ras

*KRAS* is the major mutated isoform of the Ras gene in cancers, including in ∼85% of all cancers (74, 75). Cancers driven by mutant KRAS proteins are considered refractory to most therapies. Given the “undruggable” tertiary structures of Ras, a potent and selective Ras inhibitor remained elusive for clinical use until Sotorasib was approved by the US Food and Drug Administration in May 2021 to target the growth of tumors caused by *KRAS* G12C mutation (42, 76). Nonetheless, more options are needed to target KRAS mutation-driven malignant transformation.

Surprisingly, niclosamide activated GSK-3 through disruption of the Axin-GSK3 complex (37), leading to Pan-Ras or K-Ras protein degradation (42). Ras degradation can be rescued by pharmacological GSK-3 inhibition with the GSK-3 inhibitor BIO, suggesting that niclosamide inhibits Ras signaling in a GSK-3 dependent manner. In addition, niclosamide suppresses Ras activity at various levels in colon cancer cells regardless of mutational status and inhibits G12V mutant K-Ras-induced transformation (42).

#### Stat3

Hyperactive STAT3 drives cancer progression by promoting cell proliferation, angiogenesis, migration, invasion, and immune invasion (77, 78). Hyper-activated growth factor signaling and overexpression of stimulatory receptor-ligand pairs contribute to constitutive STAT3 activation, characterized by phosphorylation of Y705 and nuclear translocation of STAT3. Specific inhibitors like ‘Stattic’ have been developed to target Y705 for STAT3 inhibition (79). However, recent studies showed that STAT3 is activated by Y727 phosphorylation independent of Y705 status in trible negative breast cancer, thereby attenuating the effect of STAT3 inhibitor ‘Stattic’ (80).

Niclosamide was identified as a potent inhibitor of STAT3 after screening 1500 clinical-approved compounds in a STAT3 reporter system (45). Niclosamide treatment inhibits phosphorylation and nuclear translocation of STAT3, leading to the repression of STAT3 transcriptional activity. Moreover, STAT3 dephosphorylation induces cell cycle arrest and apoptosis in Du145 cells expressing constitutively active STAT3 (45). More importantly, Pranay et. showed the niclosamide not only reduces the phosphorylation of the canonical site Y705 but also the phosphorylation of the non-canonical site Y727 (81). Aberrant activation of STAT3 by chemotherapeutic drugs or radiotherapy causes therapy resistance, which can be overcome by niclosamide-mediated STAT3 inhibition (46, 47, 49–51). Beyond affecting cancer cell-intrinsic signaling, niclosamide can regulate signals communicated from other cell types in the tumor microenvironment such as adipocyte-mediated epithelial to mesenchymal transition through inhibition of the interleukin-6/STAT3 signaling axis (52).

#### Notch

Like the Wnt/β-catenin pathway, Notch is also a developmental signaling pathway dysregulated in cancer that can promote cell proliferation, angiogenesis, invasion and migration, and immune evasion (82, 83). When cognate Notch ligands bind to Notch receptors, the Notch receptor is cleaved and released from the cell membrane. Subsequently, the released Notch Intracellular Domain translocates into the nucleus and regulates expression of Hes and Hey family genes such as p27cip1/waf1, p21.cyclin D1, c-Myc, Survivin, slug, and Nanog (84).

It was reported that niclosamide decreases the protein expression of Notch1, Notch2, and Notch3 in colon cancers and is associated with the inhibition of cell proliferation, repression of cell migration, and induction of apoptosis (54). Another study employed niclosamide-loaded pluronic nanoparticles (NIC-NPs) to treat thioacetamide-induced hepatocellular carcinoma (HCC) in rats (55). The researchers found that NIC-NPs treatment restores liver integrity, reduces alpha-fetoprotein (AFP) levels, and inhibits Notch signaling by reducing *notch1* mRNA levels.

#### E2F and Myc

E2Fs are the ultimate effectors of the cyclin-dependent kinase (CDK)–RB–E2F axis, the central transcriptional pathway driving cell cycle progression. Dysregulation of one or more components of this axis such as CDKs, cyclins, the CDK negative regulator, and/or the RB family of proteins is common in all cancers, leading to hyperactive oncogenic E2F activity and unrestrained proliferation (85–87). The *MYC* oncogene plays an important role in the tumorigenesis of many cancer types, is deregulated in >50% of human cancers, and is generally associated with unfavorable patient prognosis (88–91). Reported cellular functions of *MYC* include amplifying transcription of already existing gene expression programs, promoting DNA replication, increasing protein synthesis, and reprograming metabolism to support cell proliferation (90–92). Additionally, *MYC* is essential for maintaining stemness and for rewiring the tumor microenvironment to evade the immune system (91). Given the “undruggable” protein structure of the Myc protein, targeting Myc directly in cancer treatment has been a challenge for decades (89, 91).

Multiple levels of crosstalk exist between E2Fs and Myc. E2F1, E2F2 and E2F3 were shown to bind the promoter region and activate the transcription of the *MYCN* gene in *MYCN*-amplified neuroblastoma (93). Furthermore, overexpression of the Cdk-inhibitor p16^INK4A^ inhibits E2F activity, resulting in *MYCN* repression. However, overexpression of E2Fs fails to activate *MYCN* transcription in *MYCN* non-amplified neuroblastoma, indicating that E2Fs are necessary but not sufficient regulators of *MYCN* (94). In addition, *MYCN* overexpression induces E2F5 expression and promotes cell proliferation in neuroblastoma (95).

Due to the known crosstalk between E2F and Myc, we wondered whether E2F and Myc can be simultaneously targeted with a single intervention. We recently observed that a salt form of niclosamide, niclosamide ethanolamine (NEN), reduces the mRNA and protein expression of MYCN *in vitro* and *in vivo*. In line with the reduction of MYCN, MYCN target genes are globally deregulated by NEN treatment (8). NEN also reduces expression of E2F target genes. Notably, our findings are supported by another study that utilizded a secreted *Gaussia* luciferase reporter system (56) to show that niclosamide treatment reduces *MYCN* transcription.

#### NF-kB

The transcriptional factor NF-kB contributes to cancer initiation and progression, metastasis, and therapeutic resistance in human cancers (96–98). Constitutive activation of NF-kB activity caused by the inflammatory microenvironment and various oncogenic mutations are observed in many cancer types. NF-kB activation promotes cancer cell proliferation, suppresses cell apoptosis, and activates epithelial–mesenchymal transition to initiate metastasis (96, 97). Inhibition of NF-kB in tumor cells prevents tumor progression, making the NF-kB pathway an attractive therapeutic target (97). Under basal conditions, the inactive NF-κB complex (IKK, p65 and p50) is retained in the cytosol. Upon stimulation by factors such as TNFα, IκB is phosphorylated and degraded by ubiquitinylation *via* a multi-step process. The remaining NF-kB complex (p65 and p50) is then translocated into the nucleus to activate target gene transcription (99).

Niclosamide was reported to suppress NF-kB signaling and tumor growth in acute myelogenous leukemia (AML) (57, 58) and ovarian cancer (56). Mechanistically, niclosamide inhibits TNFα-mediated phosphorylation and degradation of iκbα, thereby inhibiting the phosphorylation and translocation of p65 to the nucleus (57, 100, 101). In line with the reduction of nuclear NF-kB, niclosamide represses NF-κB–mediated gene transcription as determined by luciferase reporter assays (56, 57).

#### mTOR

Mammalian/mechanistic target of rapamycin (mTOR) is a serine/threonine kinase that senses nutrients, growth factors, and environmental cues to regulate various fundamental cellular processes such as protein synthesis, autophagy, growth, metabolism, aging, and regeneration (102, 103). The mTOR pathway is frequently dysregulated in human cancers, rewiring cancer cell metabolism and the tumor microenvironment to promote tumor progression (102, 103).

Niclosamide was reported to inhibit mTOR signaling in lung cancer, ovarian cancer, cervical cancer, and the diabetic mouse kidney (61–64, 104). Accumulating evidence suggests that niclosamide-mediated mTOR inhibition may be accomplished through at least two distinct mechanisms. First, as a mitochondrial uncoupler, dissipating the mitochondrial proton gradient leads to a reduction in intracellular ATP and increase in the AMP/ATP ratio, resulting in the activation of AMP-activated protein kinase (AMPK) (8, 15, 18). AMPK activation inhibits mTOR directly through inhibitory phosphorylation of the mTORC1 subunit Raptor at Ser-792 or indirectly through disrupting the TSC2-Rheb axis (102). Second, Bruno et *al.* showed that niclosamide does not interact with or inhibit neither upstream PI3K/AKT signaling nor mTORC1 itself (59). Instead, the protonophoric activity of niclosamide is essential for dissipating protons (down their concentration gradient) from lysosomes to the cytosol and effectively lowering cytoplasmic pH, resulting in mTOR inhibition. Therefore, by suppressing mTOR signaling, niclosamide can also induce autophagy by inhibiting autophagic degradation (60).

#### HIF

Hypoxia is a common tumor microenvironment stress that induces DNA methylation (105) and generation of the oncometabolite 2-hydroxyglutarate (2-HG) (27, 29) and is associated with poor prognosis and therapeutic resistance (106).

By using a hypoxia inducible factor 1 subunit alpha (HIF1α)-based luciferase reporter system as the read-out for high-throughput screening, niclosamide was identified as an inhibitor of HIF1α signaling with an approximate IC50 of 1.59 µM (65). Niclosamide inhibits HIF1α signaling to enhance the effects of radiation in non-small cell lung cancer (67) and blocks EGF-induced HIF1α signaling to repress tumorigenesis and invasion in colorectal cancer (66). Recently, we found that NEN represses both HIF1α and HIF2α protein and HIF target genes such as PDK1, PDK3, PGK1 and LDHA in both normoxia and hypoxia (8). Because HIF-1α and HIF-2α degradation relies on α-KG-dependent prolyl hydroxylases (PHDs), which can also be inhibited by 2-HG (31), we reasoned that niclosimide-mediated HIF1α/HIF2α inhibition could result from diminished generation of 2-HG from α-KG (8).

### Tumor suppressors

In addition to inhibiting oncogenic pathways, niclosamide was also reported to activate or restore tumor suppressor signaling (**Figure 3A**, **Table 1**)

#### p53

Often referred to as “the guardian of human genome,” the p53 protein is crucial for modulating DNA repair, cell division, survival, and metabolism (107–109). Following DNA damage, p53 plays a critical role in determining whether the cell initiates the DNA repair process or induces programmed cell death to eliminate damaged DNA. By preventing cells harboring mutated or damaged genes from dividing, p53 prevents tissues from acquiring cancer fitness-promoting genomic alterations (109). While loss of wild-type p53 is common in cancer, tumor-associated p53 missense mutations can actually provide gain of function rather than simply loss of wild-type tumor-suppressing function. Mutant p53 proteins switch from a tumor suppressor to an oncogenic protein, promoting proliferation, cell survival, invasion, and metastasis (107, 108, 110).

A chemical library screen revealed that the mitochondrial uncoupling function of niclosamide selectively kills p53-deficient cells by triggering intracellular calcium flux leading to the release of arachidonic acid, a fatty acid normally detoxified by the p53 targets *ALOX5* and *ALOX12B* in wild-type cells (20). One could envision that the synthetic lethality between mitochondrial uncoupling and p53 loss would confer niclosamide tumor-suppressor functions by establishing a metabolic environment favoring the outgrowth of p53 wild-type cells. Moreover, niclosamide increases the expression of p53 at both the mRNA and protein level (8, 20). In adult cancers, TP53 is often mutated, yet in pediatric cancers such as neuroblastoma, TP53 mutations are very rare (111). Instead, p53 is typically silenced epigenetically through promoter methylation (111). Both NEN and 5-AZA treatment increase p53 protein levels in NB16 and SK-N-BE(2) cells, suggesting that mitochondrial uncoupling can upregulate p53 in NB cells through DNA demethylation.

#### AMPK

AMPK is a highly conserved central energy sensor that coordinates energy status with intracellular metabolism during cell growth, development, and adaption to stress (112). AMPK is an essential downstream effector of the tumor suppressor LKB1, which signals to COX-2 (cancer progression), ULK1/2 (autophagy), ACC1/2 (Fatty acid metabolism), mTOR (cell growth and protein synthesis), and p53 (apoptosis) (113–115).

As described before, niclosamide dissipates the mitochondrial proton gradient requisite for ATP synthesis, leading to the reduction of intracellular ATP and an increased AMP/ATP ratio, culminating in the activation of AMP-activated protein kinase (AMPK) (8, 15, 18). Additionally, niclosamide may activate AMPK through a mechanism independent of the increased AMP/ATP ratio, namely through the AMPK β2 subunit (68).

#### PP2A

Protein phosphatase 2A (PP2A) represents a family of ubiquitously expressed serine–threonine phosphatases that maintain cellular homoeostasis through regulating many important kinase-driven intracellular signaling pathways such as Akt, p53, c-Myc, and β-catenin (116, 117). The protein phosphatase 2A (PP2A) has a well-established role as a regulator of the cell cycle, signal transduction, and apoptosis. Loss of activity due to mutation in some of its subunits or the PP2A phosphatase activator (PTRA) is frequently observed in many cancer types, leading to neoplastic transformation (118, 119). In addition, CIP2A, an endogenous inhibitor of PP2A, is upregulated in many cancer cells, including non-small cell lung cancer (NSCLC) cells (120).

High-throughput screening identified niclosamide as a potent inhibitor of cancerous inhibitor of protein phosphatase 2A (CIP2A), leading to the activation of PP2A (69). The inhibitory effect of niclosamide on CIP2A depends on the reduction of CIP2A transcription, leading to lower CIP2A mRNA and protein levels and increased PP2A activity (69).

## Niclosamide regulates cellular epigenetics

DNA methylation is controlled by *de novo* methylation by DNA methyltransferases (DNMTs) and/or demethylation by DNA demethylases (121). Ten-eleven translocation (TET) DNA demethylase uses α-ketoglutarate (αKG) as the substrate to convert 5mC to 5-hydroxymethylcytosine (5hmC), followed by further reactions to remove methylation (122, 123). The two enantiomers of 2-hydroxyglutarate (2-HG) exert similar effects on TET and other α-KG-dependent dioxygenases but are generated under different conditions. The D-enantiomer (D-2-HG) is produced through gain-of-function point mutations in isocitrate dehydrogenases (IDH1/2) (124). In hypoxic tumor cells, including NB cells, the relatively lower NAD^+^/NADH ratio favors the conversion of αKG to the L-enantiomer (L-2-HG) (27, 29). Recent reports have shown that α-KG promotes pancreatic cancer and colon cancer cell differentiation through reduced DNA methylation (125, 126). However, because the hypoxic tumor microenvironment promotes the conversion of α-KG to 2-HG, preventing this metabolic reaction presents a major challenge in cancer therapy.

Although inhibitors of mutant IDH enzymes exist and are being evaluated in the clinic (some has been approved by FDA, find it out and specify), an effective therapeutic strategy to inhibit L-2-HG production remains elusive. L-2-HG is a more potent inhibitor of a-KG dependent dioxygenases (31, 127). Tumor hypoxia develops when tumor growth exceeds the ability of available vasculature to supply tumor cells with oxygen and nutrients. Clinically, tumor hypoxia is a significant obstacle to treatment because hypoxic tumor cells are more resistant to radiation therapy (128, 129) and chemotherapy (130–132). It was reported recently that DNMT inhibitor (DNMTi) treatment overcomes hypoxia-induced chemoresistance (133), suggesting that DNA hypermethylation under hypoxia can cause chemoresistance. DNA hypermethylation is reinforced through hypoxia-mediated repression of TET activity (105). Due to their similar chemical structures, 2-HG inhibits α-KG-dependent enzymes, including TET and Jumonji C domain-containing proteins (JMJDs) (31, 134), leading to hypermethylation of DNA and histones that blocks cellular differentiation. Therefore, under the low NAD^+^/NADH ratios observed in solid tumors, the potential to use α-KG as a cancer demethylation agent is limited. In addition, both D-2-HG and L-2-HG inhibit other α-KG-dependent dioxygenases such as prolyl hydroxylase domain (PHD) proteins to stabilize hypoxia inducible factor (HIF) α subunits and activate HIF signaling (27, 31) (**Figure 1**).

The signaling and metabolic alterations caused by niclosamide can potentially reprogram the global epigenetic landscape in multiple ways. On one hand, as we discovered, NEN treatment increases the intracellular NAD^+^/NADH ratio, inhibiting 2-HG generation from α-KG, leading to an increased intracellular α-KG/2-HG ratio to promote TET2 activity and DNA demethylation (8). Unlike DNMT inhibitors such 5-azacytidine, NEN treatment remodeled the DNA methylation landscape rather than simply reducing the global methylation level. The cancer epigenome is characterized with promoter CpG island hypermethylation but gene body hypomethylation. NEN treatment reversed this epigenetic remodeling pattern, reducing methylation in promoter CpG Island but increasing methylation in gene body region. This epigenetic remodeling strategy could be more effective and precise than DNMTi treatment (8). On the other hand, NEN treatment dramatically elevates ADP and AMP levels while lowering ATP levels (8). AMPK activation phosphorylates TET2 at serine 99, thereby stabilizing the tumor suppressor to promote DNA demethylation (135). Thus, it is possible that NEN treatment also increases TET activity through activating AMPK.

The fact that NEN treatment alters the cellular transcriptional profile is consistent with the theory that NEN treatment reprograms the epigenome. The number of upregulated genes is more than two-fold higher than the number of downregulated genes induced by NEN treatment, indicating that NEN treatment has a major role in activating gene expression (8). The top pathways upregulated by NEN treatment includes pathways related to neurogenesis, nervous system development, and neuron differentiation. The top downregulated pathways are involved in DNA replication and cell cycle progression. Importantly, while almost all the NEN-upregulated genes are enriched in gene signatures that indicate favorable prognosis, all the NEN-downregulated genes are enriched in gene signatures that indicate unfavorable prognosis (8). These data indicate that mitochondrial uncoupling rewires the global transcriptome in a way that leads to cell differentiation and proliferation arrest, rather than targeting one specific signaling pathway that may fail to trigger such broad-scale changes.

## Combination of niclosamide with other therapies

### Radiation

Radiotherapy is an effective cancer treatment for up to 50% of cancer patients. However, one significant challenge during radiotherapy is the buildup of acquired radioresistance (136). Thus, it is important to identify strategies that improve the efficiency of and overcome the resistance to radiotherapy.

Niclosamide was reported to enhanced the radiation sensitivity of many cancer types such as lung cancers (62, 67, 137), triple-negative breast cancer (35), nasopharyngeal carcinoma (138), and colorectal cancer (139). Synergism between niclosamide and radiotherapy may occur in part through the ability of niclosamide to inhibit multiple adaptive pathways upregulated during or following radiation. Niclosamide pretreatment induces C-Jun expression and phosphorylation, promoting apoptosis in cells that failed to control radiation-induced reactive oxygen species (ROS) (137). STAT3 was also reported to protect cells following radiation. As a potent inhibitor of STAT3, niclosamide reduces STAT3 nuclear translocation to restore radiation sensitivity (62). Niclosamide inhibits the hypoxic induction of Wnt/β-catenin and HIF1α signaling, leading to tumor radiosensitivty (35, 67). Niclosamide downregulates the expression of Ku70/80, inhibiting DNA double-strand break repair to sensitize the cancers to radiation (138, 139).

### Chemotherapy

Chemoresistance is a common obstacle to cancer treatment involving multiple resistance mechanisms (140–142). Identifying therapeutic strategies to enhance chemotherapy efficiency and overcome acquired resistance hold immense interest in the cancer biology field.

Niclosamide has shown synergistic anti-tumor effects with a broad spectrum of chemotherapy drugs. Niclosamide’s potential functions as a chemotherapy enhance are summarized in **Table 2**.

**Table 2:** Niclosamide has synergetic effect with chemotherapy.

Drugs	Cancer type	Potential mechanism	Reference
cytarabine	Acute Myelogenous Leukemia	NS	(57)
etoposide		NS	
daunorubicin		NS	
dasatinib	chronic myeloid leukemia	inhibiting Erk/Mnk1/eIF4E pathway	(143)
castration	prostate cancer	inhibition ofandrogen receptor variants	(144)
cisplatin	renal cellcarcinoma	NS	(145)
	lung cancer	Suppression of lung resistance−related protein and c−myc	(146)
	esophageal cancer	Inhibition of STAT3 pathway	(147)
	Hepatocellular Carcinoma	Inhibition of STAT3 pathway	(53)
Oxaliplatin	Colorectal Cancer	increased H_2_O_2_ production	(148)
5-FU	esophageal cancer	Inhibition of STAT3 pathway	(147)
paclitaxel	triple negative breast cancer	NS	(149)
	esophageal cancer	Inhibition of STAT3 pathway	(147)
erlotinib	colorectal cancer	Inhibition of STAT3 pathway	(49)
SN38	colorectal cancer	Inhibition of STAT3 pathway	(50)
Doxorubicin	Breast Cancer	downregulating the Wnt/β-catenin pathway	(150)
camptothecin	glioblastoma	NS	(151)

NS, not sure.

### Immunotherapy

Discoveries from the last decade have shown that immunotherapy, unleashing power from the patient’s own immune system to recognize and eliminate cancer cells, is a promising approach for cancer treatment. The immune receptor/ligand pair PD-1/PD-L1 constitutes a key inhibitory immune checkpoint system hijacked by cancer to escape destruction by the immune system, thereby highlighting its importance as a target for cancer immunotherapy (152). Niclosamide is reported to disrupt PD-1/PD-L1 interactions in non-small cell lung cancer (153), metastatic lung adenocarcinoma (154), and pancreatic cancer (41, 155) primarily through PD-L1 ligand downregulation in cancer cells. Importantly, several studies observed that niclosamide potentiates PD-1/PD-L1 blockade in preclinical cancer models (41, 153–155). At the molecular level, this reduction of PD-L1 expression by niclosamide may rely on the suppression of STAT3 phosphorylation and transcription factor binding to the PD-L1 promoter in the nucleus (153).

## Clinical trials

The plethora of preclinical studies demonstrating impressive antiviral and anticancer effects of niclosamide have led to a series of clinical trials. There are currently 31 records of clinical trials involving niclosamide, as published on the clinicaltrials.gov database. Among these, 16 trials relate to Covid-19 treatment and 8 trials relate to cancer treatment. We summarize the cancer-relevant clinical trials in **Table 3**. Despite the promising data generated in preclinical models, proof of efficacy and safety is still required. These properties are associated with diverse biopharmaceutical challenges such as the relationship between physicochemical properties and oral absorption of the drug with clinical outcomes (156). Published data regarding the pharmacokinetics (PK) of niclosamide suggest that it has poor oral bioavailability (11), potentially limiting its application as a cancer drug, consistent with observations made in clinical trial NCT02532114 (156). In this trial, either 500mg or 1000mg niclosamide was given three times daily to patients. However, the maximal plasma concentration ranged from 35.7–82 ng/mL (0.1µM-0.25 µM), a range that failed to be consistently above the minimum effective concentration in preclinical studies (156). In contrast, the ongoing clinical trial NCT02807805 is administering 1200 mg of reformulated orally bioavailable niclosamide orally (PO) three times daily to patients, resulting in 0.21µM-0.723 plasma niclosamide concentrations exceeding the therapeutic threshold of > 0.2 µM. In prostate cancer patients, combination of niclosamide with abiraterone/prednisone induced a prostate-specific antigen (PSA) response in 5 of 8 evaluable patients (158). Overall, niclosamide displays an excellent safety profile across these clinical trials. However, the bio-availability and standalone anti-tumor effect of niclosamide are still major challenges. To overcome these limitations, new delivery strategies and rational combination therapies with other treatments need to be developed.

**Table 3:** The clinical trials using niclosamide for cancer therapy.

Ref	Cancer type	Potential target	Mechanism	Phase
NCT05188170	Acute Myeloid Leukemia	CREB (58)	Inducing apoptosis and cell cycle arrest	Phase 1
NCT04296851	Familial adenomatous polyposis (FAP)	Axin-GSK3 (37)	inhibition of Wnt pathway and Snail-mediated EMT	Phase 2
NCT03123978	Metastatic/Recurrent Prostate Carcinoma	IL6-Stat3-AR pathway (46)	overcome enzalutamide resistance and inhibit migration and invasion	Phase 1
NCT02807805	Metastatic/Recurrent Prostate Carcinoma	androgen receptor variant 7	synergizes with abiraterone	Phase 2
NCT02687009	Colon Cancer	Frizzled receptor (36)	Inhibition of Wnt/β-catenin pathway	Phase 1
NCT02532114	Castration-Resistant Prostate Carcinoma		Inhibition of androgen receptor splice variants or Wnt/β-catenin pathway (156)	Phase 1
NCT02519582	Colorectal Cancer	Wnt/β-catenin pathway signaling (157)	restricting S100A4-driven metastasis	Phase 2

## Conclusion and future directions

Cancer is the second leading cause of death in the world after heart disease, accounting for 1 out of every 6 deaths in 2021 (159). An effective and low-risk cancer treatment has remained elusive for decades. Indeed, current treatments such as chemotherapy, radiation therapy, hormone therapy, and immunotherapy each have their own limitations as a “magic bullet” against cancer. Namely, their off-target effects stem from the fact that these therapies are aiming at the “passengers” but not the “drivers” in the cancer cell “bus.”

A major metabolic hallmark of cancer is to divert glucose flux away from mitochondrial oxidation to cytosolic fermentation and lactate production, a process also known as the Warburg effect (160, 161). According to Warburg himself, the consequence of this metabolic reprogramming is to convert differentiated normal cells to undifferentiated cells, namely, cancer cells (4, 162). Hence, identifying compounds that can target the metabolic reprogramming of cancer should present substantial benefit for cancer treatment. Recently, we found that the mitochondrial uncoupler niclosamide could reverse this metabolic hallmark of cancer, leading to a rewiring of the global epigenetic landscape and the induction of cell differentiation (8). Thus, we propose the mitochondrial uncoupler niclosamide can serve as a compound to target cancer metabolic reprogramming.

Numerous oncogenic pathways or tumor suppressors have been reported to be influenced by niclosamide treatment. However, these alterations could be secondary effects resulting from inhibition of the primary target. What could this primary target be? Three major targets of niclosamide have been proposed. Firstly, as a mitochondrial uncoupler, niclosamide uncouples the mitochondrial membrane potential from ATP synthesis (11). Secondly, the protonophoric activity of niclosamide can dissipate protons from lysosomes (59). Thirdly, niclosamide directly binds to GSK3, resulting in disruption of the Axin-GSK3 complex and attenuation of canonical Wnt activity (37). Among these targets, the mitochondrial uncoupling function is reported to be essential for targeting both p53 wild-type and mutant cancers (20). Nonetheless, additional studies are needed to elucidate the primary target of niclosamide as an anti-tumor compound.

Clinically, the major challenge for niclosamide is poor oral bioavailability, potentially limiting its use as a cancer drug (11, 156). Efforts have been taken to improved its bioavailability, including: (1) reformulating niclosamide for better delivery and stability (158, 163–166) and (2) modifying the structure of niclosamide to generate derivatives with enhanced efficiency (167, 168) or pharmacokinetics (169, 170). Nonetheless, the process of identifying these derivatives involved screens with readouts of either cell apoptosis or oncogenic pathway inhibition, processes that may not reflect the primary property of niclosamide as anti-tumor compound, thereby reinforcing the need to identify the primary target of niclosamide to accelerate pharmacological development of new derivatives. Another area of important need is to improve the clinical potential of niclosamide; specifically, initiating studies that address the synthetic lethality of niclosamide in cancer to identify pathway dependencies or gene mutations sensitive to niclosamide treatment. Based on the results of clinical trials, it is likely that niclosamide treatment alone will not be enough to achieve a complete response in cancer patients. Therefore, further effort is needed to test combination therapies using niclosamide with other therapeutic agents.

## Author contributions

HJ, AL and JY conceived and wrote the manuscript. All authors contributed to the article and approved the submitted version.

## Funding

This work was supported by a Stanford Maternal and Child Health Research Institute Research Scholar Award (2020) and an American Cancer Society Research Scholar Grant (RSG-20-036-01) to JY.

## Conflict of interest

HJ and JY submitted a patent application related to this manuscript.

The remaining authors declare that the research was conducted in the absence of any commercial or financial relationships that could be construed as a potential conflict of interest.

## Publisher’s note

All claims expressed in this article are solely those of the authors and do not necessarily represent those of their affiliated organizations, or those of the publisher, the editors and the reviewers. Any product that may be evaluated in this article, or claim that may be made by its manufacturer, is not guaranteed or endorsed by the publisher.
